# Sequencing and annotation of the complete mitochondrial genome of a threatened labeonine fish, *Cirrhinus reba*

**DOI:** 10.5808/GI.2020.18.3.e32

**Published:** 2020-09-10

**Authors:** Mohammad Nazrul Islam, Shirin Sultana, Md. Jobaidul Alam

**Affiliations:** 1 Department of Biotechnology, Sher-e-Bangla Agricultural University, Dhaka 1207, Bangladesh; 2 Fisheries Biotechnology Division, National Institute of Biotechnology, Dhaka 1349, Bangladesh; 3 Department of Fisheries, Ministry of Fisheries and Livestock, Dhaka 1000, Bangladesh; 4 Interdisciplinary Program of Biomedical, Mechanical and Electrical Engineering, Pukyong National University, Busan 48513, Korea

**Keywords:** complete mitogenome, Illumina sequencing, mitochondrial DNA, reba carp, threatened species

## Abstract

The mitochondrial genome of a species is an essential resource for its effective conservation and phylogenetic studies. In this article, we present sequencing and characterization of the complete mitochondrial genome of a threatened labeonine fish, *Cirrhinus reba* collected from Khulna region of Bangladesh. The complete mitochondrial genome was 16,597 bp in size, which formed a circular double-stranded DNA molecule containing a total of 37 mitochondrial genes (13 protein-coding genes, 2 ribosomal RNA genes, and 22 transfer RNA genes) with two non-coding regions, an origin of light strand replication (OL) and a displacement loop (D-loop), similar structure with other fishes of Teleostei. The phylogenetic tree demonstrated its close relationship with labeonine fishes. The complete mitogenome of *Cirrhinus reba* (GenBank no. MN862482) showed 99.96% identity to another haplotype of *Cirrhinus reba* (AP013325), followed by 90.18% identity with *Labeo bata* (AP011198).

## Introduction

The reba carp, *Cirrhinus reba* (Hamilton, 1822) is an important freshwater labeonine fish of the family Cyprinidae and the Order Cypriniformes. The species is geographically distributed over the Indian sub-continent including Bangladesh, India, Nepal, and Pakistan [1]. In Bangladesh, it is locally known as reba carp, tatkini, raik or bhagna bata and the natural habitats of the fish include the rivers, small creeks, natural depressions, and floodplains [2]. Siltation, conversion of wetlands, drying-up, and fragmentation of habitats, aquatic pollution, over fishing and climate changes have posed an alarming threat to the existence of many fish species including *C. reba*. Consequently, among 54 threatened freshwater fishes, *C. reba* is categorized as a near threatened (NT) species in Bangladesh [3]. In India, *C. reba* has been reported as vulnerable species based on International Union for Conservation of Nature (IUCN) criteria [4]. A few reports on the induced-breeding [5], stock identification [2], cryogenic preservation of sperm [1] have potentiated for the hatchery-based selective breeding, identification of the population status and the conservation of *C. reba*.

The mitochondrial genome is composed of a circular, double-stranded DNA with semi-autonomous central dogma machinery and situated in the mitochondria of each vertebrate cell outside the nuclear genome [6]. It possesses 37 genes in specific order that code for 13 polypeptides necessary for the oxidative phosphorylation (OxPhos) system [7]. The mitochondrial DNA of most metazoan species is predominantly inherited maternally [8]. The clonal inheritance, coupled with a substitution rate is typically 5–10 times more than that of nuclear DNA [9]. Mitochondrial DNA is an essential raw material as molecular marker for the investigation of population genetics [10], phylogenetic and evolutionary histories [11], genetic barcoding, and biodiversity of species [12], genetic disorder or mitochondrial diseases [13].

In the present study, we characterized the complete mitochondrial genome of *C. reba* using next-generation sequencing (NGS) with Illumina MiSeq platform. Sequence of the full mitochondrial genome would be an essential source to design specific molecular markers, for instance, single nucleotide polymorphisms studying population genetic structure. Moreover, detailed decoding of the mitochondrial genome would be a valuable insight for resolving any conservation and management strategies of the culture promising but NT fish.

## Methods

### Specimen collection, genomic, and mitochondrial DNA isolation

The specimen was collected from Khulna, Bangladesh (22°50′44.3″N, 89°32′27.7″E) in March 2017. The specimen is stored at the Department of Biotechnology, Sher-e-Bangla Agricultural University, Sher-e-Bangla Nagar, Dhaka 1207, Bangladesh. The genomic DNA was extracted from the fish tissue using the DNeasy Blood and Tissue Kit (Qiagen, Hilden, Germany) according to the manufacturer’s manual. The mitochondrial partial COI sequence was amplified for the species confirmation using the BCL-BCH primers [14]. The specimen was confirmed based on both morphological characteristics and its COI sequence with 100% identity to the GenBank database (GenBank no. AP013325). The mitochondrial DNA was extracted with a commercially available mitochondrial DNA isolation kit (Abcam, Cambridge, UK) followed by the manufacturer’s protocol and DNA concentration was checked by qubit fluorometer.

### Library construction and NGS

In advance to the library preparation, the purified mitochondrial DNA was fragmented as 300–350 bp by Covaris M220 Focused-Ultrasonicator (Covaris Inc., Woburn, MA, USA). A library was prepared by TruSeq RNA library preparation kit V2 (Illumina, San Diego, CA, USA) which needed a couple of steps including end repair reaction, adenylation at 3ʹ ends, adaptor ligation, and enrichment of DNA fragments by PCR. To have better quality DNA, the enriched DNA library templates were purified using DNA purification kit (RBC Bioscience, Jerusalem, Israel) and quantified using qubit fluorometer. Size, purity, and quality of the constructed DNA libraries were further validated by the 2100 Bioanalyzer (Agilent Technologies, Santa Clara, CA, USA). Finally, the NGS was performed using the Illumina MiSeq platform 2×300 bp pair ends (Illumina).

### Sequence assembly and annotation of the mitochondrial genome

The MiSeq raw reads were assembled using the Geneious Prime 2020.0.3 software [15] to construct a complete mitogenome of *Cirrhinus reba*. The gene order, sequences, and sizes of each of the 13 protein-coding genes and 2 ribosomal RNA genes were organized with the ORF finder program (https://www.ncbi.nlm.nih.gov/orffinder/) following a reference mitogenome of *Cirrhinus reba* (GenBank no. AP013325). All transfer RNA genes were identified, the positions of their anticodons were predicted by using the ARWEN software [16].

### Gene mapping and phylogenetic tree construction

The circular gene map of *C. reba* was drawn by OGDRAW software (https://chlorobox.mpimp-golm.mpg.de/OGDraw.html). The phylogenetic tree was constructed by MEGA7 program using the Minimum Evolution (ME) algorithm [17] performing with 1,000 bootstrap replications.

## Results and Discussion

### Genome organization

**Fig. 1.**
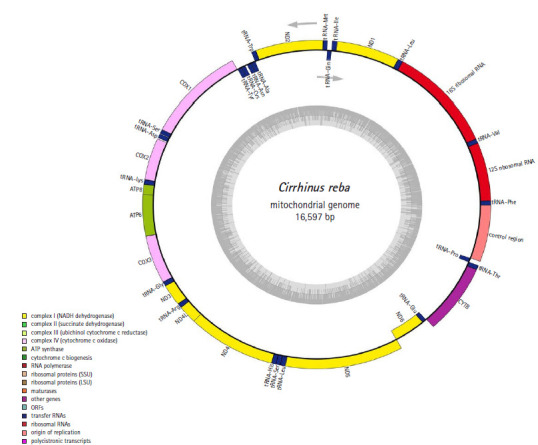
Gene mapping and organization of mitochondrial genome of the *Cirrhinus reba*.

The complete mitochondrial genome of *C. reba* (GenBank no. MN862482) is a closed circular molecule of 16,597 bp in size. It possesses the typical combination of 37 genes including 13 protein-coding genes, 22 tRNA genes, two genes for ribosomal RNA subunits (12SrRNA and 16SrRNA), and two non-coding regions (control region, D-loop, and origin of light strand, OL) (Table 1, Fig. 1). Twenty-eight genes were located on the heavy (H) strand, while the ND6 gene and eight tRNA genes were transcribed from the light (L) strand (Fig. 1).

The overall nucleotide composition of *C. reba* mitogenome was found biased toward A + T contents (58.93%, A = 32.91% and T = 26.02%) over the G + C contents (41.07%, G = 15.16% and C = 25.91%) indicating a strong anti-guanine bias commonly observed in fishes [18]. This pattern was almost the same as the Cyprinid species, which has 15.70% of G content [19]. A total of 31 bp overlapping region was found in 11 different locations of the *C. reba* mitogenome. Besides, 11 intergenic spacers were present in the mitogenome involving a total size of 34 bp. The longest spacer included a nucleotide sequence of 15 bp which located between the tRNA Asp and *COX2* genes. As shown in Table 1, the positive (+) numbers indicate intergenic space/gap between genes, the negative (–) numbers indicate overlapping between genes, and the zero (0) revealed either overlap or space does not exist between genes in the *C. reba* mitogenome.

### Protein-coding genes

The mitochondrial genome of *C. reba* is consisted of 13 canonical protein-coding genes (PCGs) of 11,412 bp in length, accounted for 68.76% of the total mitogenome. With exception to the reading frame of ND6 gene, which was oriented clock-wise direction on the light strand, reading frames of all other PCGs were oriented counter-clock wise direction on the heavy strand (H). The shortest PCG was ATP8 (165 bp), whereas the longest one was ND5 (1,824 bp). Except for the COX1 gene with an unusual and alternative start codon, GTG (Valine), other twelve PCGs started with the typical translation initiation codon, ATG (Methionine). The open reading frame of ATP8 used TAG as stop codon, whereas, the remaining seven PCGs (*ND1, COX1, ATP6, COX3, ND4L, ND5*, and *ND6*) terminated with the typical stop codon (TAA), as in other vertebrates [20]. Four PCGs, *COX2*, *ND3, ND4, CYTB* ended with an incomplete termination codon (T--), while the ND2 gene ended with “TA-” (Table 1). These incomplete stop codons are assumed to be completed by using the posttranscriptional polyadenylation, poly-A tail [21]. These kinds of variations in the termination codons are not uncommon among vertebrate mitochondrial genes and previously reported in other bony fishes [22].

Analysis of the sequences of 13 PCGs showed that overall percentage of A and T contents (58.93 %) also reflected in the codon usage of PCGs where the first, second and third position of the codons estimated the A plus T contents as 49.6%, 58.9%, and 69%, respectively (Table 2). The G content (15%) of all PCGs presents obvious anti-guanine characteristics as similar to other bony fishes [23]. Here, we observed that the 2nd and 3rd position of the codons were dominated by T (40.4%) and A (46.7%) contents, respectively over the C and G contents (Table 2). A very strong bias against the guanine contents (4.9%) was also demonstrated at the wobble or third position of codons across all 13 PCGs.

### Ribosomal RNA and transfer RNA genes

The mitogenome of *C. reba* possesses two genes encoding two ribosomal RNA subunits, a small (12S) and a large (16S) which were typical as in other mitogenomes. Both the ribosomal RNA genes were located on the H-strand and consisted of 15.91% (2,641 bp) of the total circular mitogenome. The 12S rRNA (946 bp in length) located between the tRNA^Phe^ and tRNA^Val^, whereas the 16S rRNA was 1,695 bp in length and was located between tRNA^Val^ and tRNA^Leu^. The A + T content (55.24%) was higher than the G plus C content (44.76%) in the ribosomal RNAs where the nucleotide, adenine was more prevalent (35.25%) followed by cytosine (24.50%) as similar to the previous reports in other bony fishes [24].

The twenty-two tRNA genes were identified in the mitochondrial of *C. reba* which demonstrated a varied size of 67–78 bp in length, estimating a total length of 1,580 bp (~9.6% of the total mitogenome) (Table 1). Among all tRNA genes, eight tRNAs were located on the L-strand, whereas the remaining fourteen tRNA genes were on the H-strand (Fig. 1). The highest content of A and T was observed in tRNA^Ala^ and tRNA^His^ (69.6%), whereas that of the lowest content was observed in tRNA^Met^ (42%) which was consisted of previously published reports on other teleosts [24]. All these 22 tRNA genes were predicted to capable of folding typical cloverleaf secondary structures and showed great similarity as it is in other vertebrate mitogenomes [23].

### Non-coding regions

Like other animal mitochondrail genome, *C. reba* contained no introns and possessed two non-coding regions, an OL and a control region or displacement loop (D-loop) region. The OL region, consisted of 34 nucleotides was situated between tRNA^Asn^ and tRNA^Cys^ and was oriented on the L-strand in a cluster of five tRNA genes (WANCY region). D-loop region of *C. reba* consisted of 930 nucleotides which represented 5.6 % of the total mitogenome. The control region was overpresented by A plus T content (67.6 %). The AT-rich D-loop contains promoters and an origin of replication of mtDNA which are essential for transcription and replication of mtDNA, respectively [25]. The D-loop region is very flexible to size variation.

### Phylogenetic relationship analysis

**Fig. 2.**
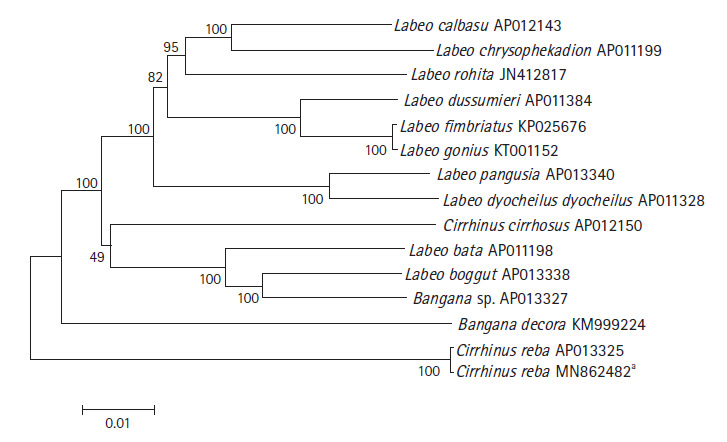
Phylogenetic tree of *Cirrhinus reba* in the subfamily Labeoninae. The phylogenetic relationship was analyzed by MEGA7 program with Minimum Evolution (ME) algorithm and 1,000 bootstrap replications. GenBank accession number of each species is shown next to its scientific name.** Reported in the present study.

The phylogenetic relationship of *C. reba* with other labeonine fishes was constructed using 13 complete mitochondrial genomes of the subfamily Labeoninae by MEGA 7.0 program. The mitogenome of our species of interest, *C. reba* (GenBank no. MN862482) showed 99.6% sequence identity with different haplotype of *C. reba* (GenBank no. AP013325, deposited from Japan), followed by 90.18% identity with *Labeo bata* (AP011198). Slight variations between two haplotypes of *C. reba* might reflect different populations of the same species. However, two haplotypes of *C. reba* was clearly isolated from other 11 species of labeonine fishes and formed a separate clade (Fig. 2). Two close relatives of *C. reba, L. pangusia*, and *C. cirrhosis* have already been considered globally as NT and vulnerable species, respectively [3]. Therefore, we alarmingly recommend further studies relating to conservation of the labeonine fish including reba carp.

## Figures and Tables

**Table 1.** Organizational characteristics of the complete miotochondrial genome of *Cirrhinus reba*

Gene/element	Strand	Nucleotide position		Size (bp)	Codon		Anti-codon	Intragenic nucleotides	A + T (%)
		From	To		Start	End			
tRNA^Phe^	H	1	70	70	-	-	GAA	0	52.9
12S rRNA	H	71	1024	954	-	-		0	51.0
tRNA^Val^	H	1025	1096	72	-	-	TAC	0	50.0
16S rRNA	H	1097	2783	1687	-	-		0	57.6
tRNA^Leu^	H	2784	2860	77	-	-	TAA	0	48.1
ND1	H	2861	3835	975	ATG	TAA		3	58.6
tRNA^Ile^	H	3839	3912	74	-	-	GAT	–3	54.1
tRNA^Gln^	L	3910	3980	71	-	-	TTG	1	60.6
tRNA^Met^	H	3982	4050	69	-	-	CAT	0	42.0
ND2	H	4051	5096	1046	ATG	TA-		–1	59.6
tRNA^Trp^	H	5096	5167	72	-	-	TCA	1	63.9
tRNA^Ala^	L	5169	5237	69	-	-	TGC	1	69.6
tRNA^Asn^	L	5239	5311	73	-	-	GTT	0	52.1
OL	-	5312	5345	34	-	-		0	52.9
tRNA^Cys^	L	5346	5412	67	-	-	GCA	1	52.2
tRNA^Tyr^	L	5414	5483	70	-	-	GTA	1	48.6
COX1	H	5485	7035	1551	GTG	TAA		1	58.3
tRNA^Ser^	L	7037	7105	69	-	-	TGA	4	53.6
tRNA^Asp^	H	7110	7181	72	-	-	GTC	15	62.5
COX2	H	7197	7887	691	ATG	T--		–1	59.6
tRNA^Lys^	H	7887	7964	78	-	-	TTT	0	55.1
ATP8	H	7965	8129	165	ATG	TAG		–7	61.8
ATP6	H	8123	8806	684	ATG	TAA		–1	61.4
COX3	H	8806	9591	786	ATG	TAA		0	55.9
tRNA^Gly^	H	9592	9663	72	-	-	TCC	0	66.7
ND3	H	9664	10012	349	ATG	T--		0	59.3
tRNA^Arg^	H	10013	10082	70	-	-	TCG	0	51.4
ND4L	H	10083	10379	297	ATG	TAA		–7	55.6
ND4	H	10373	11753	1381	ATG	T--		0	59.1
tRNA^His^	H	11754	11822	69	-	-	GTG	–1	69.6
tRNA^Ser^	H	11822	11892	71	-	-	GCT	–1	59.2
tRNA^Leu^	H	11892	11966	75	-	-	TAG	2	60.0
ND5	H	11969	13792	1824	ATG	TAA		–4	62.2
ND6	L	13789	14310	522	ATG	TAA		–1	57.7
tRNA^Glu^	L	14310	14380	71	-	-	TTC	4	62.0
CYTB	H	14385	15525	1141	ATG	T--		0	60.1
tRNA^Thr^	H	15526	15598	73	-	-	TGT	–4	47.9
tRNA^Pro^	L	15595	15667	73	-	-	TGG	0	57.5
Control region (D-loop)	-	15668	16597	930	-	-	-	0	67.6

**Table 2.** Base composition of PCGs of *Cirrhinus reba* mitochondrial genome

	Base composition (%)					Total
	T	C	A	G	A + T	
PCGs						
*ND1*	27.2	27.7	31.4	13.7	58.6	975
*ND2*	24.0	29.3	35.6	11.1	59.6	1,046
*COX1*	30.0	24.8	28.3	16.8	58.3	1,551
*COX2*	27.5	25.3	32.1	15.1	59.6	691
*ATP8*	26.7	26.7	35.2	11.5	61.8	165
*ATP6*	30.0	25.7	31.4	12.9	61.4	684
*COX3*	25.8	28.4	30.0	15.8	55.9	786
*ND3*	30.4	27.2	28.9	13.5	59.3	349
*ND4L*	28.6	29.3	26.9	15.2	55.6	297
*ND4*	26.5	27.9	32.6	13.0	59.1	1,381
*ND5*	27.5	26.0	34.7	11.8	62.2	1,824
*ND6*	44.1	11.1	13.6	31.2	57.7	522
*CYTB*	28.0	26.2	32.2	13.7	60.1	1,141
Position in the codon						
1st	24.2	24.3	25.4	26.1	49.6	3,807
2nd	40.4	27.0	18.6	14.0	58.9	3,803
3rd	22.3	26.1	46.7	4.9	69.0	3,802
Across all PCGs	28.9	25.8	30.2	15.0	59.2	11,412

Reverse complementary sequence was used for ND6 gene which was encoded on the L-strand. PCG, protein-coding gene.
